# Near-infrared tunable metalens based on phase change material Ge_2_Se_2_Te_5_

**DOI:** 10.1038/s41598-019-41859-x

**Published:** 2019-03-29

**Authors:** Wei Bai, Ping Yang, Jie Huang, Dingbo Chen, Jingjing Zhang, Zhaojian Zhang, Junbo Yang, Bing Xu

**Affiliations:** 1 The key laboratory of Adaptive Optics, Chinese Academy of Sciences, Chengdu, Sichuan 610209 China; 2 Center of Material Science, National University of Defense Technology, Changsha, 410073 China; 3 Institute of Optics and Electronics, Chinese Academy of Sciences, Chengdu, Sichuan 610209 China; 4 University of Chinese Academy of Sciences, Beijing, 100049 China

**Keywords:** Metamaterials, Micro-optics

## Abstract

Metasurfaces draw everyone’s attention because they can precisely control the phase, amplitude and polarization of emergent light to achieve light field control in recent years. As one of the most practicable devices among the many applications of metasurface, metalens can extremely reduce the size as well as complexity of optical systems and realizes the higher optical quality compared with conventional lens. So it will be very potential to use metalens in integration systems to reaching higher integration and efficiency. In addition, dynamic control is always desirable in optical systems. In this work, we firstly design a near-infrared tunable metalens treating phase change materials as the meta-atoms which makes the tunable metalens become more compact. At designed wavelength of 1.55 μm, the focusing efficiency of our amorphous metalens is more than 16 times of the efficiency when it works at crystalline state, and its focal length can stay almost unchanged when the GST state is switched. The broadband performance of the metalens is also confirmed. This work may bring some good opportunities for the revolution of the next generation tunable integrated optics.

## Introduction

Flexible and portable devices becomes more and more popular as people enjoys more convenient life with the help of modern technology. Integrated systems are indispensable in such devices. However current integrated devices need to be improved because they still use gratings or tapered fibers to achieve optical connection which are inefficient.

Metasurfaces are a class of 2D materials that are unable to obtain in nature world. Owing to their ability to easily control of light field, they have stir up a spree of research in the last decades. Metasurfaces are composed of some well-designed subwavelength scatters which can manipulate the phase, amplitude and polarization of light in nano-scale resolution^1–39^. As the processing technology develops, metasurfaces can be fabricated using conventional nano-manufacturing techniques, which is an important step towards practicable application. New design method such as Deep Learning^1^ is also studied to promote the development of Micro-nano optics. Recently, various work such as focus^2,3^, deflect^4^, cloak^5^, hologram^6^, nonlinear^7^, vortex beam^8^ and computing^9,10^ based on metasurfaces have been reported. Among all of these functions, focus is absolutely an exciting and important direction because not only metalens has much smaller volume compared with conventional bulky lens but also it exhibits better optical performances^2,3,11^. The proposal of conformal metasurfaces even make the metalens become more flexible in the applications^12,13^. Considering there already are a lot of work concentrated on metalens to optimize its functions, like chromatic aberration correction^14–18^, multi-focus^19,20^, focal distance tuning^21,22^, and ultra-broadband focusing^23^, metalens is an ideal approach to replace gratings to achieve light interconnection in optoelectronics or all optical systems. Sometimes switching on and off is necessary in the optical systems, and previous work on large-scale efficiency tuning with a single device is rarely reported, so it is meaningful to develop metalens which has the function of switch.

The popular ways to achieve dynamic control of metasurfaces are utilizing stretchable structure^24–26^ to fix structure parameters or 2D materials^21,22,27–30^ like graphene, sulfide to fix materials parameters. However there is little work discussed the efficiency tuning because they can hardly control the absorption of structure in the Near-infared. Phase change materials are absolutely a good choice for tuning materials because of their significant complex permittivity variance between crystalline state and amorphous state^40,41^. Among them, the chalcogenide compound Ge_2_Se_2_Te_5_ (GST) which has remarkable fast switching speed (nanosecond or less)^42^, high switching robustness (potentially up to 10^15^ cycles)^43^, is one of the most prevalent phase change materials. Typically, its state can be easily changed by optical, electrical or thermal energy. When staying at the temperature between transition temperature and melting temperature, the amorphous GST will be crystallized, and it will be back to amorphous phase after a short high density laser pulse melts and quick quenches. All the methods of phase change have been experimentally demonstrated^44–46^. Lately, more and more researchers have great interests in using phase change materials in nanophotonic devices. A great deal of functions about tunable mid-infrared plasmonic chiral metamaterial and resonance mode^31,32^, controlling lateral Fano interference optical force^33^, beam steering^34,35^ and multifunctional metasurfaces^36,37^, based on phase change materials have been investigated. All these studies have offered a promising future for active metasurfaces.

In this article, we firstly design the efficiency large-scale tunable transmittive metalens using phase change materials as the meta-atoms. The metalens is composed of a group of well-arranged GST nanocubes, and the abrupt phase change generates from Pancharatnam-Berry (P-B) phase shift. The Numerical Aperture (NA) of the metalens is as high as 0.71. The focusing efficiency of the amorphous metalens at designed wavelength, 1.55 μm, is about 16 times of the crystalline metalens. At the same time, the focal length of amorphous metalens and crystalline metalens are almost equal. Compared with existing devices such as a silicon metalens +tunable filter, our tunable metalens is more compact and fits the request of nanophotonics. Although the contrast ratio of the proposed device is smaller than existing devices, it can be greatly improved by fixing the structure parameters making polarization conversion efficiency of the crystalline GST close to zero. Moreover, our metalens can focus from 1.49 μm to 1.65 μm, as a result of P-B phase shift, while its tuning ability remains strong. This research expands the functions of metalens further, making it more powerful at tuning in the near-infrared wavelength range, which will bring great influences on compact optical systems such as photoelectrical integrations or all-optical integrations.

## Methods

**Figure 1:**
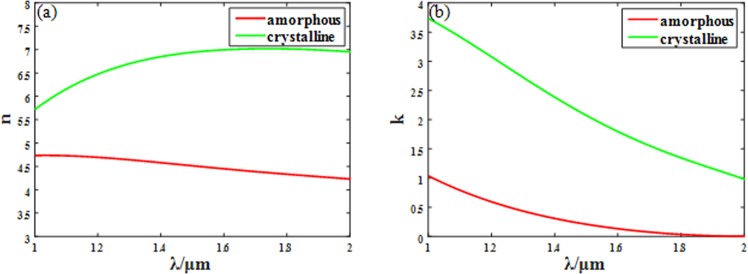
Complex refractive index variance of GST. (**a**) The real part *n* versus wavelength (**b**) The imagery part *k* versus wavelength for both amorphous state and crystalline state.

Phase change material GST is firstly proposed for re-writeable optical data storage^42^ because of its reversible transition between amorphous and crystalline states. Different states correspond to different lattice configurations, amorphous, metastable Face centered cubic (Fcc) and the stable hexagonal phase, which leads to different complex permittivity and refractive index. Figure 1(a and b) show the large difference of complex refractive index of GST between the amorphous and crystalline states at wavelength *λ* = *1~2* μm^46^. Especially, the complex refractive index of GST changes from *n*_*amorphous*_ = 4.48 + 0.16i to *n*_*crystalline*_ = 6.96 + 1.93i at the wavelength of 1.55 μm, which is big enough for efficiency tuning of metalens. When light propagates in a dielectric, the propagation formula of light field can be written as:

1 $${\boldsymbol{E}}={{\boldsymbol{E}}}_{{\boldsymbol{0}}}{e}^{i{k}_{0}x}={{\boldsymbol{E}}}_{{\boldsymbol{0}}}{e}^{i\omega (n+ik)x/c}={{\boldsymbol{E}}}_{{\boldsymbol{0}}}{e}^{\alpha x}{e}^{iwnx/c}$$

The absorption coefficient $\alpha =-\,\omega k/c$, so the imaginary part of the complex refractive index has a huge influence on the absorption of the dielectric, and the obvious change of refractive index along with the state change will give GST remarkable tuning ability.

There are two main ways to achieve phase control using metasurfaces in recent years: the propagation phase and the P-B phase. The later can get precise phase via rotating the angel between the nanostructure and the *x* axis. Let’s suppose that a left/right circularly polarization incident light (LCP/RCP) is vertical to the structure where its orientation direction forming an angle *θ* with *x* axis. By applying the jones matrix, the output light field can be expressed as:

2 $$\begin{array}{ccc}{{\boldsymbol{E}}}_{out} & = & \alpha [\begin{array}{cc}co{s}^{2}\theta  & sin\theta cos\theta \\ sin\theta cos\theta  & si{n}^{2}\theta \end{array}]{{\boldsymbol{E}}}_{L/R}\\  & = & \alpha [\begin{array}{cc}co{s}^{2}\theta  & sin\theta cos\theta \\ sin\theta cos\theta  & si{n}^{2}\theta \end{array}]\,[\begin{array}{c}1\\ \pm i\end{array}]\\  & = & \frac{1}{2}\alpha (cos2\theta +i\ast sin2\theta )[\begin{array}{c}1\\ \mp i\end{array}]+\frac{1}{2}\alpha [\begin{array}{c}1\\ \pm i\end{array}]\\  & = & \frac{1}{2}\alpha {e}^{i\ast 2\theta }{{\boldsymbol{E}}}_{R/L}+\,\frac{1}{2}\alpha {{\boldsymbol{E}}}_{L/R}\end{array}$$

Here, ${{\boldsymbol{E}}}_{L/R}={{\boldsymbol{e}}}_{x}\pm i\ast {{\boldsymbol{e}}}_{y}$ only distinguishes the polarization of the light, and the coefficient $1/\sqrt{2}$ is negligible, $\alpha$ represents the propagation constant. From the formula, the incident LCP state light can be divided into two different LCP/RCP states due to the fact that the co-polarization light only has amplitude modulation and the counter-polarization light can have $\phi =2\theta$ phase modulation simultaneously. With the angle rotating from 0 to π, the abrupt phase change can cover the range from 0 to 2π, which is enough for focusing. However, the size of the unitcell need to be designed specifically to achieve the effect that its focusing efficiency is high for amorphous GST but low for crystalline GST.

The phase distribution for metalens focusing should satisfy the formula:

3 $${\rm{\phi }}(r,f)=-\,{k}_{0}(\sqrt{{r}^{2}+{f}^{2}}-f)$$

Where ${k}_{0}=2\pi /\lambda$ is the free space wave vector, *r* is the coordinate of each unitcell, and *f* is the focal length. Considering we can get any abrupt phase change $\phi (r,\,f)$ at any position via rotating the unitcell at an angle of $\phi (r,\,f)/2$, once the size of the unitcell is determined, the next step is just rotating them to satisfy equation (3).

**Figure 2:**
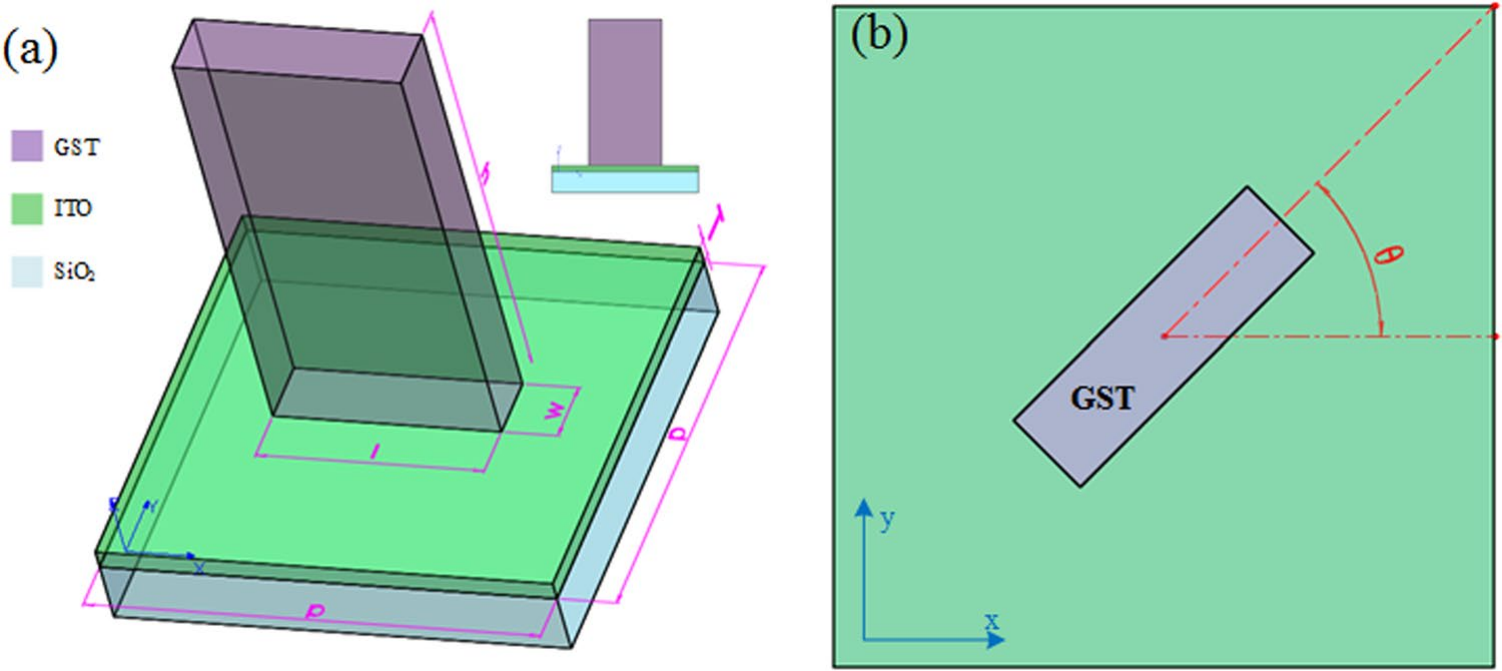
Structure of unitcell. (**a**) The overview of unitcell, nanocuboid GST is settled on ITO conductive layer and substrate SiO_2_, periodic *p* = 0.7 μm, GST height *h* = 0.7 μm, length *l* = 0.35 μm, width *w* = 0.1 μm. ITO film thickness T = 0.03 μm. The insert is the side view of the unitcell. (**b**) The overlook of the unitcell, the orientation direction of structure have an angle *θ* with *x* axis.

The unit-cell is depicted in Fig. 2(a). The nanocuboid waveguide GST is settled on a 30 nm thin film indium tin oxide (ITO) conductive layer and SiO_2_ substrate with its orientation direction having an angle of *θ* with *x* axis as shown in Fig. 2(b). The performance of the unitcell is mainly controlled by the four parameters: periodic, height, length and width. In our design, the values of parameters are determined to be *p* = 0.7 μm, *h* = 0.7 μm, *l* = 0.35 μm and *w* = 0.1 μm after optimization at wavelength 1.55 μm. The high aspect ratio structure can be fabricated with Atomic Layer Deposition (ALD) method which has been experimentally proven before^2,38^. As a transparent electrode, the ITO layer owns sufficient conductivity to allow electrical Joule heating of GST, so the state of GST can be switched reversibly by applying electrical current pulse through the conductive layer. These methods provide the feasibility for experimental demonstration in the future.

## Results

**Figure 3:**
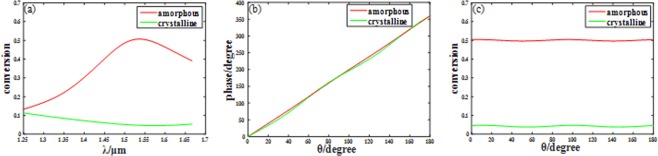
(**a**) The conversion efficiency from LCP to RCP of unitcell versus wavelength for both amorphous GST and crystalline GST. (**b**) The phase modulation versus rotating angle *θ* for both states at designed wavelength 1.55 μm. (**c**) The conversion efficiency from LCP to RCP versus rotating angle *θ* for both states at 1.55 μm.

The simulation of the unitcell is performed by using finite-element method in frequency domain with the unitcell boundary in xy direction and the open boundary in z direction. The minimum edge length is 7.4 nm. The incident light source LCP planewave propagates along +z direction through the substrate. According to theoretical analysis, a part of light will be turned into RCP with abrupt phase change of *2θ*. The conversion efficiency from LCP to RCP for two different GST states is illustrated in Fig. 3(a). Although the conversion efficiency are not very high for both GST states due to the absorption of materials (50% for amorphous and 5% for crystalline at the wavelength of 1.55 μm), a big contrast ratio (about 10 times) is required. The big variance of conversion efficiency makes the structure own extraordinary regulating capability at 1.55 μm. Actually, the structure has a good tuning ability in a wide range from 1.49 μm to 1.65 μm, which can be extended to broadband imaging. Then the *2θ* phase modulation for both GST states structure are validated, and the result is characterized in Fig. 3(b). The simulation shows that the phase change linearly covers the whole 2π range with the angle changed from 0 to π, however the rotating has little influence on conversion efficiency of the structure as shown in Fig. 3(c). All simulations have demonstrated that the unitcell will be functional for metalens converging and efficiency tuning even for switching on and off.

**Figure 4:**
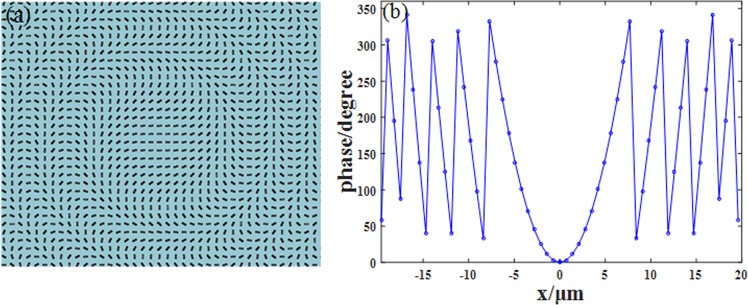
(**a**) The overlook of the metalens, the unitcell is arranged with its angle satisfying $\theta =\phi (r,\,f)/2$. (**b**) The real phase distribution of the metalens with sampling at the center of each unitcell.

As the designed unit-cell performs well, the structure need to be arranged properly according to equation (3). The overall structural layout is illustrated in Fig. 4(a). The metalens’ radius is 20 μm; focal length is 20 μm, and NA is 0.71 at the designed wavelength of 1.55 μm. Then the phase distribution versus radius *r* sampling at the center of each unitcell is calculated. The curve in Fig. 4(b) shows the imperfect phase distribution which is inevitable in the design process, thus the final focusing result will deviate slightly from theory. Although the result can be further improved by designing unitcell with smaller periods, the coupling between each unitcell will disturb the phase distribution which is unacceptable in metalens.

**Figure 5:**
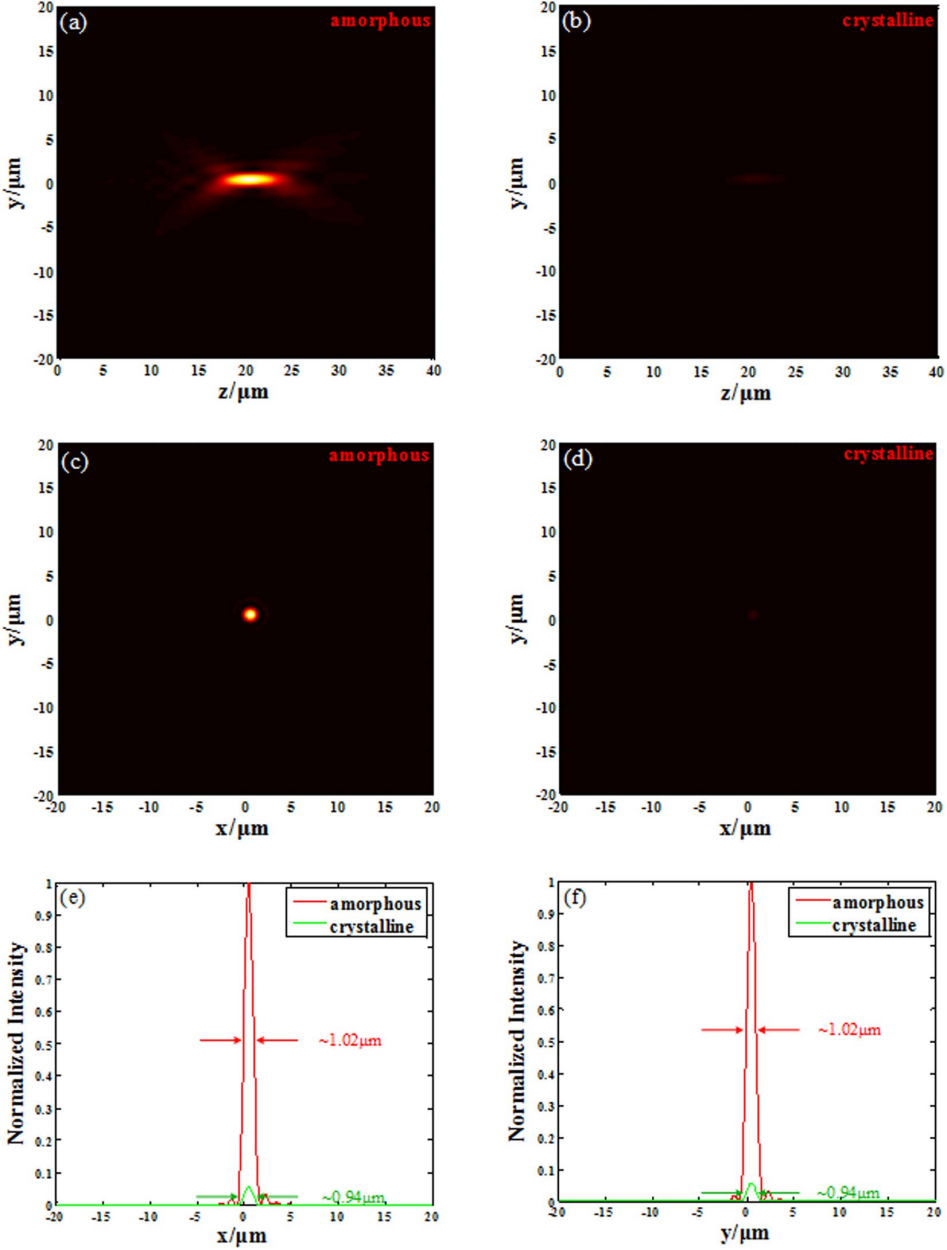
Focusing contrast for two GST states at designed wavelength 1.55 μm. (**a**) The light field distribution along *z* axis for amorphous metalens. (**b**) The light field distribution along *z* axis for crystalline metalens. (**c**) The light intensity in focal plane for amorphous metalens. (**d**) The light intensity in focal plane for crystalline metalens. (**e**) The FWHM of the spot in focal plane for *x* direction. (**f**) The FWHM of the spot in focal plane for *y* direction.

The results of metalens are simulated by using finite integrity in time domain with open boundary for all direction, and the size of the uniform mesh is set as 20 nm along all axes to minimize numerical errors. The light field for amorphous metalens along z axis is shown in Fig. 5(a). Its focal length is 20.4 μm with its Depth of Focus (DOF) of 5.0 μm. As discussed before, the slight difference (${\rm{\Delta }}f=0.4\,\mu m,\,2 \%$) comes from the phase discontinuity and that can be accepted. The *x-y* plane light intensity at the focal plane is given in Fig. 5(c), and the position of the spot in focal plane is also influenced by the discontinued phase $({\rm{\Delta }}x=\,{\rm{\Delta }}y=0.55\,\mu m)$. The intensity normalized full width at half maximum (FWHM) of the spot in focal plane for x and y direction are shown in Fig. 5(e and f) using red lines. The result (FWHM = 1.02 μm, less than $\lambda /2NA$) indicates that the spot in focal plane is symmetrical and it overcomes the diffraction limit due to the subwavelength manipulating of the light field. The focusing efficiency of amorphous metalens is about 32.82%. It is reasonable because the amorphous GST also has little absorption at the designed wavelength of 1.55 μm. The focusing efficiency is defined as the ratio of the light intensity of the focal plane to the light intensity of the incident plane.

Then, the phase change material GST is changed from amorphous state to crystalline state with structure parameters remain unchanged. Nevertheless the thickness decreasing of GST (6%)^46^ along with state change is considered. All the simulation process are same with amorphous metalens. The light intensity in y-z plane is depicted in Fig. 5(b). Obviously, the intensity has a big contrast with intensity distribution of amorphous metalens. It is imaginable because the conversion efficiency of unitcell from LCP to RCP decreases significantly when the GST is changed from amorphous to crystalline. The focal spot can hardly be seen, which demonstrated that the metalens’ efficiency can be greatly tuned via the state change of GST. The crystalline metalens’ focal length is 20.6 μm with its DOF of 5.0 μm. The little deviation of focal length compared with amorphous metalens comes from the imperfect phase modulation in Fig. 3(b) marked in green line. Figure 5(d) shows the *x-y* plane light filed at the focal plane, and the aberration of the spot is same with amorphuse metalens $({\rm{\Delta }}x=\,{\rm{\Delta }}y=0.55\,\mu m)$. The FWHMs of the spot in focal plane for *x* and *y* directions are both 0.94 μm which is shown in Fig. 5(e and f), as a comparison in green line. More importantly, the focusing efficiency of crystalline metalens is 2.01%, which is just about 1/16 of the amorphous metalens. The result proves that our metalens has great efficiency tuning ability while its other performances are not disrupted. The tunable metalens can even be used for switch on and off considering its converging efficiency is only 2.01%, which can be ignored in most cases.

## Discussion

**Figure 6:**
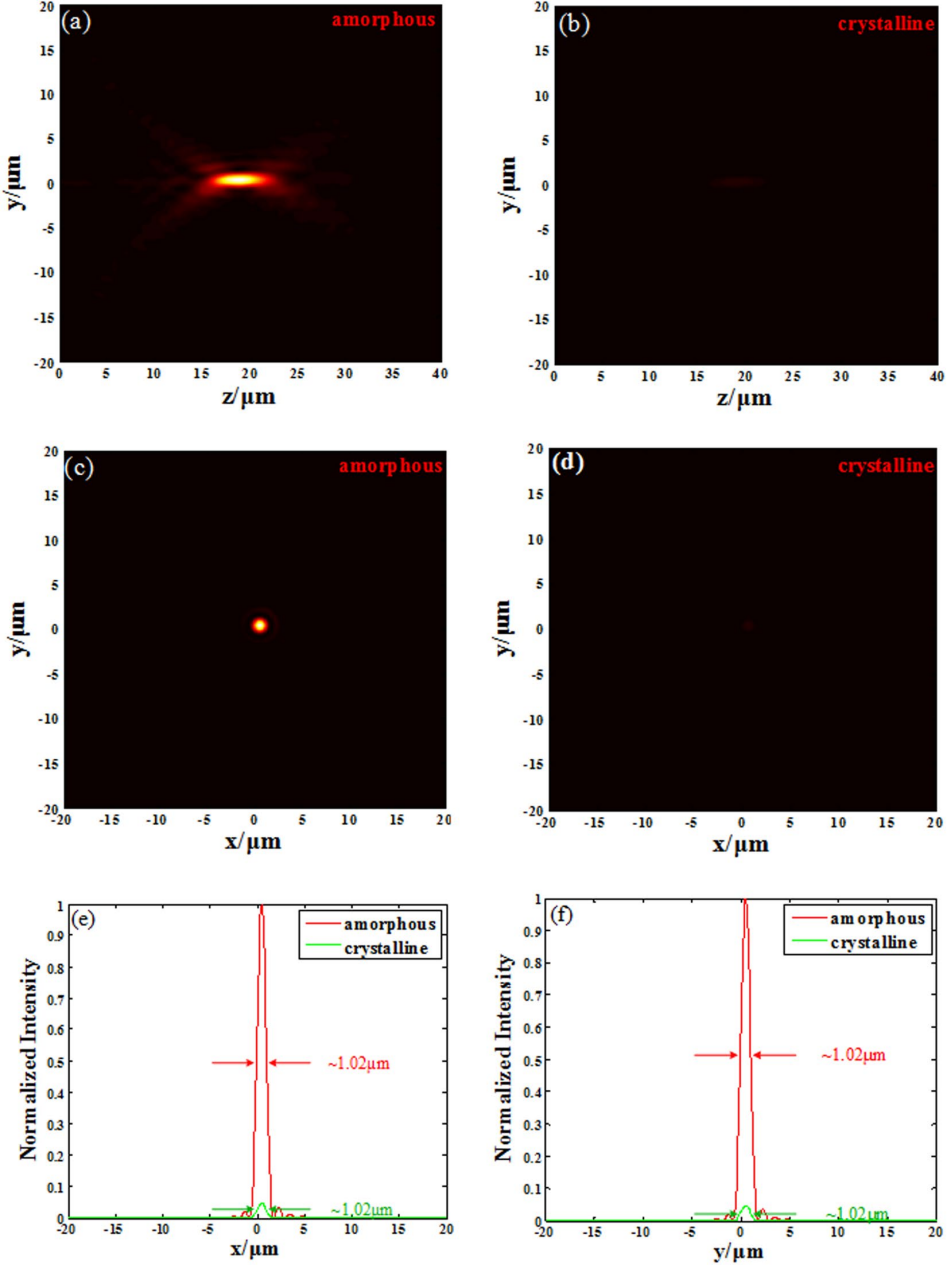
Focusing contrast for two GST states at designed wavelength 1.65 μm. (**a**) The light field distribution along *z* axis for amorphous metalens. (**b**) The light field distribution along *z* axis for crystalline metalens (**c**) The light intensity in focal plane for amorphous metalens. (**d**) The light intensity in focal plane for crystalline metalens. (**e**) The FWHM of the spot in focal plane for *x* direction. (**f**) The FWHM of the spot in focal plane for *y* direction.

It is well known that the P-B phase shift operates in the broadband range. We have mentioned that our unitcell has excellent tuning ability in the range from 1.49 μm to 1.65 μm, so the metalens’ broadband focusing function shall be verified by operating the metalens designed for 1.55 μm at other wavelengths in this range. Firstly, the light source is turned into 1.65 μm LCP, making the metalens separately working at amorphous state and crystalline state. Its light field along z axis is shown in Fig. 6(a and b). The results demonstrate that the metalens still has excellent tuning ability when it works at 1.65 μm without any adjustment, but its focal length has turned from 20.4/20.6 μm to 18.8/18.8 μm with DOF equals 4.9/4.9 μm. That is reasonable because of its chromatic aberration character, and it can be corrected by methods like doublet metalens^11,39^ etc. The x-y plane light intensity is also depicted in Fig. 6(c and d), and the positions of the spots are both ${\rm{\Delta }}x=\,{\rm{\Delta }}y=0.55\,\mu m$ for the two states. As shown in Fig. 6(e and f), the spots in focal plane are both symmetrical with their FWHMs are 1.02/1.02 μm, which still maintains the subwavelength resolution ability. The focusing efficiency of the metalens is 28.41% and 1.43% for the amorphous state and the crystalline state respectively, and its contrast ratio is even higher.

**Figure 7:**
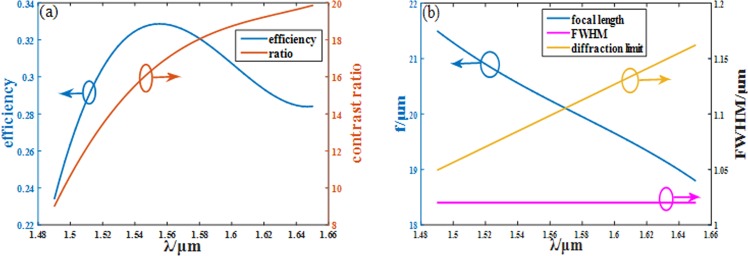
(**a**) The efficiency of amorphous metalens (blue line) and the contrast ratio between two GST states (orange line) versus wavelength. (**b**) The focal length (blue line), FWHM (purple line) and diffraction limit (yellow line) versus wavelength for amorphous metalens.

When the incident light is changed into 1.49 μm LCP, the focal length of the metalens turns into 21.5/21.6 μm in the amorphous state and the crystalline state. The DOF is 5.1/5.1 μm, and the spot in focal plane stay at the same position in x-y plane with the FWHM of the symmetrical spot in focal plane is 1.02/1.02 μm. The focusing efficiency of the metalens is 23.39% and 2.60% for amorphous state and crystalline state. The amorphous metalens’ efficiency is almost 10 times higher than the crystalline metalens’ although the tuning range becomes smaller. Figure 7 shows the amorphous metalens’ performance in the whole broadband range. The above results prove that our metalens can be used for broadband focusing only with small change of focal length, which is normal and can be corrected while its tuning ability remains powerful.

In this work, we employ phase change material GST to tune the efficiency of metalens. Meanwhile, it can be used to achieve many other functions like focal length or position tuning by designing unitcell properly. Since different metalens parameters indicate different phase distribution according to equation (3) (which may needs some deformation), various tuning functions can be realized by arranging unitcell appropriately to make the phase change between amorphous state and crystalline state fitting the variance.

## Conclusion

As a kind of phase change material, GST was only used in rewriteable data medium firstly, but we combine it with popular metalens due to its obvious parameter (permittivity) adjustment and control between the amorphous state and the crystalline state. As the scatter itself, the GST unitcell utilizes P-B phase shift to satisfy the phase distribution for metalens. The metalens designed at a wavelength of 1.55 μm can focus at the focal plane (2% error), overcoming the diffraction limit for both states, and the focusing efficiency of the amorphous metalens is about 16 times of that for the crystalline metalens providing excellent tuning ability even for switching. Then we demonstrate that the metalens carries on the broadband operation from P-B phase, so it can focus in a wide range from 1.49 μm to 1.65 μm with superior tuning ability simultaneously. Other functions such as focal length and position tuning with GST are also investigated. Our work proves the practicability of GST metalens, and it may make a great contribution in the integration of optical system.

## Data Availability

The data of material GST are available from the ref. ^46^.
