# Author Correction: Near-infrared tunable metalens based on phase change material Ge_2_Sb_2_Te_5_

**DOI:** 10.1038/s41598-020-60082-7

**Published:** 2020-02-19

**Authors:** Wei Bai, Ping Yang, Jie Huang, Dingbo Chen, Jingjing Zhang, Zhaojian Zhang, Junbo Yang, Bing Xu

**Affiliations:** 1 The key laboratory of Adaptive Optics, Chinese Academy of Sciences, Chengdu, Sichuan 610209 China; 2 Center of Material Science, National University of Defense Technology, Changsha, 410073 China; 3 Institute of Optics and Electronics, Chinese Academy of Sciences, Chengdu, Sichuan 610209 China; 4 University of Chinese Academy of Sciences, Beijing, 100049 China

Correction to: *Scientific Reports* 10.1038/s41598-019-41859-x, published online 29 March 2019

The original version of this Article contained an error in the title and Introduction of the paper where “Ge_2_Sb_2_Te_5_” was incorrectly given as “Ge_2_Se_2_Te_5_”.

As a result, in the Introduction,

“Among them, the chalcogenide compound Ge_2_Se_2_Te_5_ (GST) which has remarkable fast switching speed (nanosecond or less)^42^, high switching robustness (potentially up to 10^15^ cycles)^43^, is one of the most prevalent phase change materials.”

now reads,

“Among them, the chalcogenide compound Ge_2_Sb_2_Te_5_ (GST) which has remarkable fast switching speed (nanosecond or less)^42^, high switching robustness (potentially up to 10^15^ cycles)^43^, is one of the most prevalent phase change materials.”

These errors have now been corrected in the PDF and HTML versions of the Article.

